# Hidden blood loss between percutaneous pedicle screw fixation and the mini-open Wiltse approach with pedicle screw fixation for neurologically intact thoracolumbar fractures: a retrospective study

**DOI:** 10.1186/s13018-023-03581-3

**Published:** 2023-02-16

**Authors:** Haitao Jiang, Wenbo Sheng, Hantao Yuan, Jianhua Xu, Xiaochun Chen, Xiaohua Gu, Sibo Li

**Affiliations:** Department of Spine Surgery, Seventh People’s Hospital of Shanghai University of Traditional Chinese Medicine, Shanghai, China

**Keywords:** Thoracolumbar fractures, Percutaneous pedicle screw fixation, Mini-open Wiltse approach with pedicle screw fixation, Hidden blood loss

## Abstract

**Background:**

The aim of this study was to determine the proportion of hidden blood loss (HBL) in patients treated with minimally invasive surgery, and to compare the HBL between patients treated with percutaneous pedicle screw fixation (PPSF) and the mini-open Wiltse approach with pedicle screw fixation (MWPSF).

**Methods:**

From January 2017 to January 2019, a total of 119 patients with thoracolumbar fractures were included in the analysis, of which 58 cases received PPSF and 61 cases received MWPSF. The clinical information and demographic results were collected and compared. And the HBL of the patients is calculated by the combination formulas of Nadler, Gross and Sehat.

**Results:**

Compared with the PPSF group, operation time of MWPSF is shorter. The fluoroscopy times are 13.6 ± 3.0 in PPSF group and 5.6 ± 1.6 in MWPSF group (*p* < 0.001). As shown in Table 3, the intraoperative blood loss in PPSF group is 31.9 ± 9.6 ml, which is significantly less than that in the MWPSF group (44.0 ± 14.9 ml). The HBL (445.7 ± 228.9 ml), and HBL% (91.2 ± 7.7%) of the PPSF group are significantly higher than that in the MWPSF group (*P* < 0.05). And the total blood loss (TBL) of the PPSF group (477.6 ± 228.8 ml) is also more than that in the MWPSF group (401.0 ± 171.3 ml).

**Conclusions:**

Our results suggest that in the minimally invasive surgical treatment of thoracolumbar fractures, the perioperative HBL is much higher than visible blood loss (VBL). Although PPSF has less intraoperative blood loss, it has higher TBL and HBL than those of MWPSF. Compared with MWPSF, we should pay more attention to the postoperative anemia status of patients with thoracolumbar fractures undergoing PPSF surgery.

## Background

Spinal fractures are common fractures, among which the thoracolumbar spine fractures have the highest incidence [1, 2]. Thoracolumbar fractures are sometimes accompanied by nerve damage, and for this type of thoracolumbar fractures, the conventional open surgical approach is still a preferred option [3, 4]. Nerve compression can be relieved with adequate exposure and effective decompression, thereby facilitating the recovery of patients [5]. However, traditional open surgery often leads to longer incisions and more severe soft tissue injuries, which may lead to chronic pain and limited motion [6, 7]. Therefore, minimally invasive surgery may be a better choice for neurologically intact thoracolumbar fractures [8–10]. Currently, there are mainly two minimally invasive surgical approaches—the percutaneous pedicle screw fixation (PPSF) and the mini-open Wiltse approach with pedicle screw fixation (MWPSF). Compared with the traditional open surgical approach, the minimally invasive approach can reduce the damage of soft tissues, intraoperative blood loss, postoperative pain, and lead to better postoperative functional recovery [3, 8–11]. Our hospital has been treating neurologically intact thoracolumbar fractures through minimally invasive surgery since 2015. In clinical practice, we noticed that despite short operative time and little intraoperative visible blood loss (VBL), the postoperative anemia still occurred. We speculated that perioperative hidden blood loss (HBL) may explain this phenomenon. The concept of HBL was first proposed by Sehat, which refers to blood loss caused by diffusion into tissues, residual in dead space, or hemolysis [12]. Multiple studies have shown that HBL is an important component of total blood loss during orthopedic surgery [13–15]. During the minimally invasive surgery, we often ignore HBL because of the short operation time and little intraoperative VBL.

The aim of this study was to determine the proportion of HBL in patients treated with minimally invasive surgery, and to compare the HBL between patients treated with PPSF and MWPSF.

## Methods

Our study has been reviewed and approved by our hospital's Medical Ethics Committee, and informed consent has been obtained from all patients. After reviewing the medical records of all patients with thoracolumbar fractures from January 2017 to January 2019, a total of 119 patients were included in the analysis, of which 58 cases received PPSF and 61 cases received MWPSF. Inclusion criteria were: (1) age > 18 years, with skeletal maturity; (2) fresh single-segment thoracolumbar (T10–L2) fracture [AO Spine type A3]; (3) intact neurological function; (4) complete medical and imaging data were available. Exclusion criteria were: (1) history of spinal surgery; (2) pathological or osteoporotic vertebral fractures; (3) multiple fractures; (4) severe hematologic disease; (5) administration of antiplatelet drugs or non-steroidal anti-inflammatory drugs (NSAIDs) one month before admission.

### Operative techniques of PPSF group

After general anesthesia, the patient lay prostrate on the radiolucent operating table, the chest and the anterior superior iliac spine are cushioned, and the abdomen is suspended. Then, the vertebral pedicle projections of the injured vertebral body and the adjacent upper and lower vertebral bodies were obtained by C-arm, and the K-wires, parallel and vertical to the connecting line of spinous processes, were used for positioning on the body surface. The skin of the surgical area was disinfected. With the marked points as the center, the longitudinal incisions, about 1.5–2 cm in length, were made. The skin, subcutaneous tissue and lumbar dorsal fascia were incised, hemostasis was conducted, and blunt dissection was performed from the space between the multifidus muscle and the longissimus, then the articular process of the vertebral body could be. Under the guidance of C-arm, the tip of the puncture needle was placed on the outer edge of the projection of the pedicle of vertebral arch (10 o'clock on the left and 2 o'clock on the right), inclined 10° to 15°, and puncture into the vertebral body parallel to the endplate. Fluoroscopy was performed to confirm that the tip of the puncture needle did not break through the medial cortex, and lateral fluoroscopy was performed to confirm that the puncture needle was parallel to the endplate, about 1 cm away from the anterior edge of the vertebral body. The inner core was pulled out and the guide wire was placed in. The expanding tube and protective sleeve were introduced step by step through the guide wire. The screw channel was expanded and the pedicle screw was screwed into the vertebral body through the guide wire, and the guide wire was then taken out. C-arm was performed to confirm that the screws were well positioned. All pedicle screws were placed sequentially. The connecting rod was pre-bent and inserted into the tail groove of the pedicle screw through the subcutaneous muscle channel. The nuts were screwed and tightened in turn. C-arm was performed to confirm that the height of the injured vertebra was restored and the placement of the screw rod was satisfactory. The incision was sewed up after rinsing.

### Operative techniques of MWPSF group

The preoperative preparations were the same as in the PPSF group. The positions of fractured vertebral body and the adjacent upper and lower vertebral bodies were located and marked with the help of C-arm. The skin in the surgical area was disinfected. A posterior median longitudinal incision was made with the fractured vertebral body as the center and the subcutaneous tissues were separated, then the lumbar dorsal fascia was longitudinally cut. The space between longissimus muscle and the multifidus was bluntly separated, and the articular process of the vertebral body and transverse processes were exposed. The entry point (the intersection of the central axis of the transverse process and the outer edge of the superior articular process) was determined, incline 10° to 15°, a pedicle opener was used to slowly drill into the pedicle, and the depth was measured after reaching the appropriate position. A screw tap was used to expand the nail channel. The pedicle screws with appropriate length were implanted in turn. C-arm fluoroscopy was performed to confirm that the screws were well positioned. Two connecting rods with appropriate length were placed, then the nuts were screwed and tightened in turn. C-arm was performed again to confirm that the height of the injured vertebra was restored, and the placement of the screw rod was satisfactory. The incision was sewed up after rinsing.

**Fig. 1:**
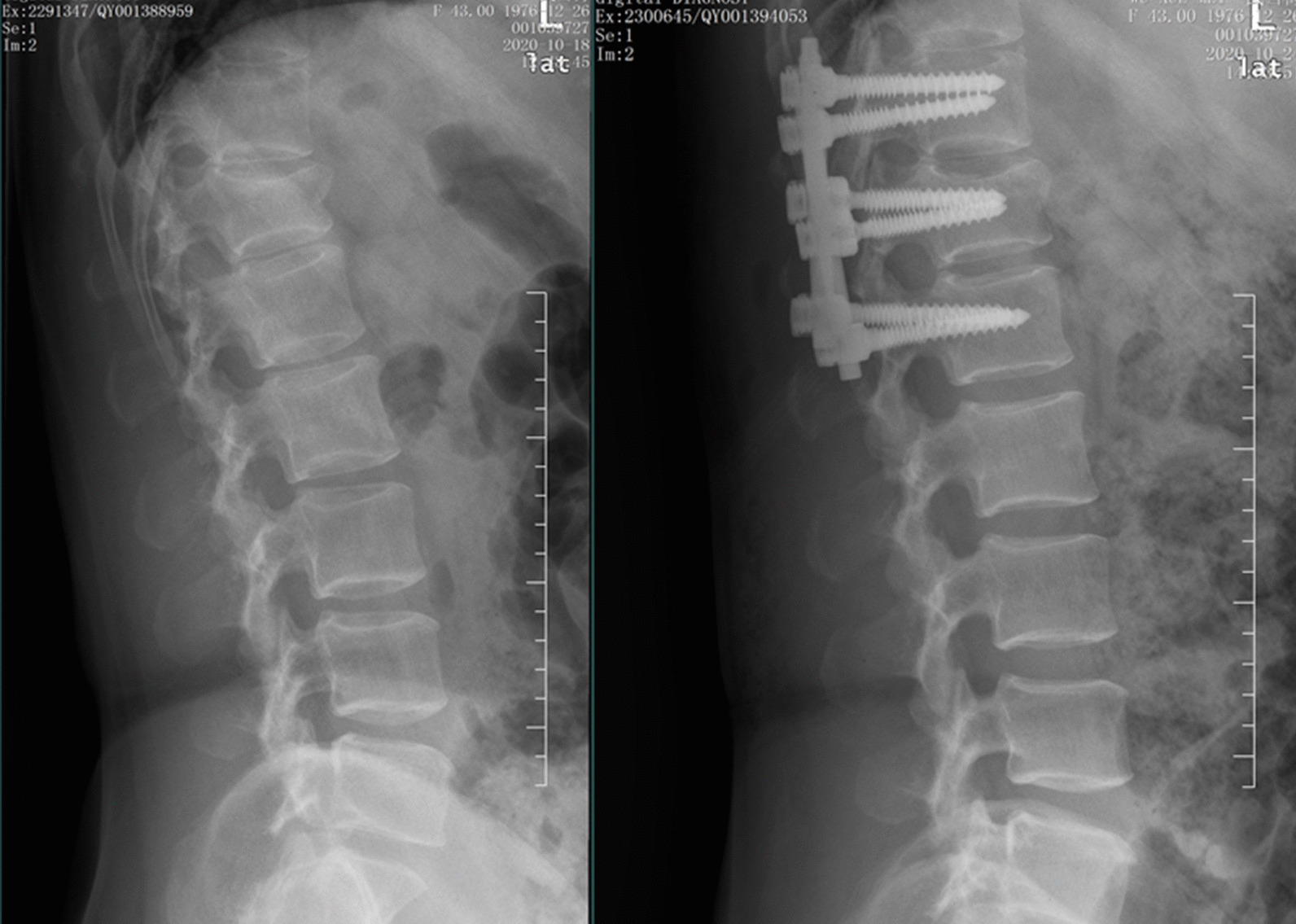
The preoperative and postoperative X-ray of PPSF group

**Fig. 2:**
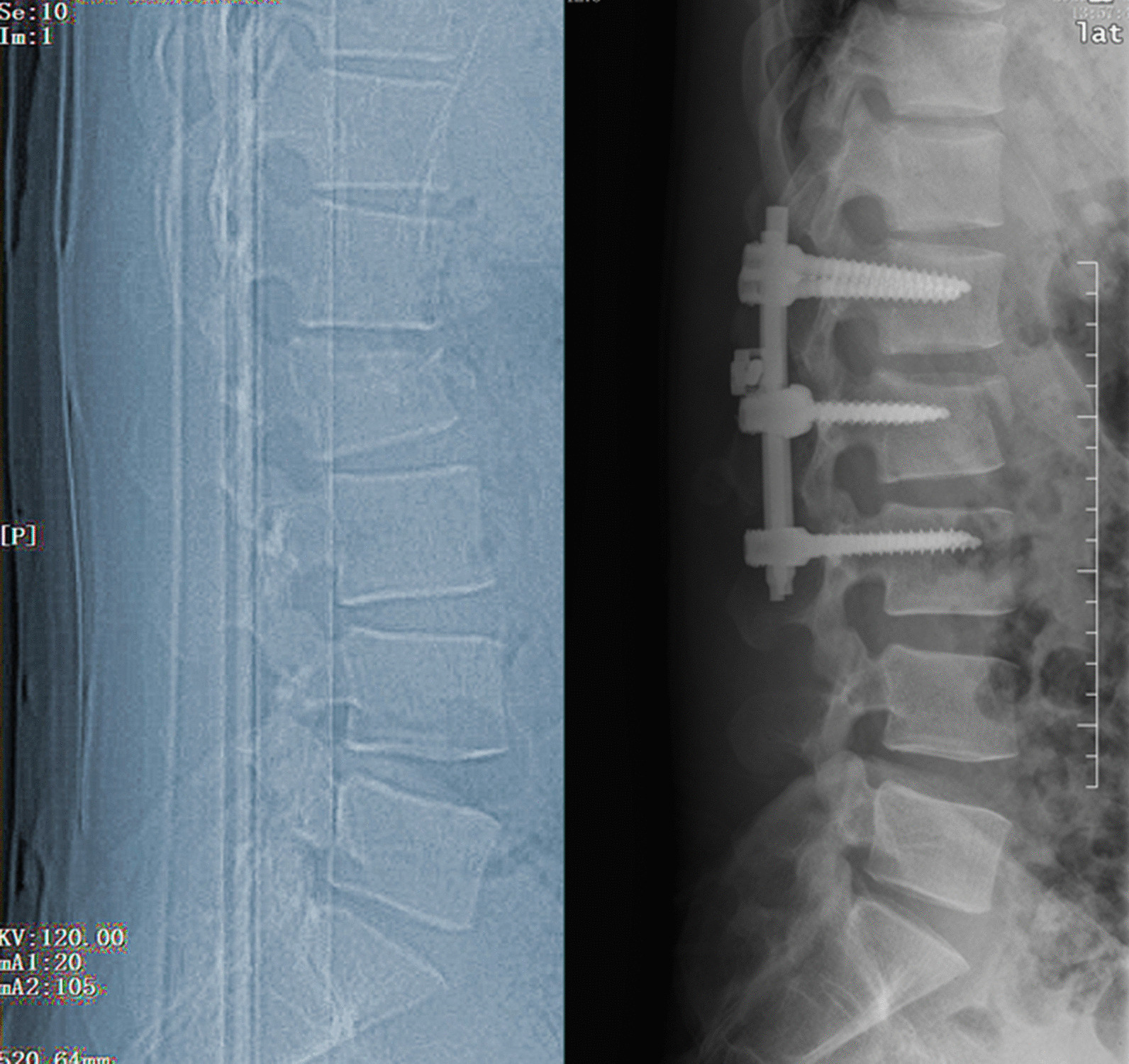
The preoperative and postoperative X-ray of MWPSF group

Routine anti-infection, analgesia, detumescence and other symptomatic treatments were given after surgery, and wound dressings were changed regularly. On the third day after the operation, the patients were instructed to perform low back muscle exercises on the bed. The sutures on the incision were removed at 12–14 days. Moderate off-bed activities were performed under the protection of the waist circumference at 4 to 6 weeks after the operation. The patients were regularly followed up at our hospital (Figs. 1, 2).

### Data collection

Before the operation, preoperative variables were evaluated and recorded, including age, sex, height, weight, and fracture segment. Data postoperatively included the duration of the operation, the length of the incision, the number of fluoroscopies, blood loss, and the length of hospital stay after the operation. During the operation, anesthesiologists recorded the amount of blood loss, including the blood in the suction bottles and the blood in the weighted swabs. All patients had a hemoglobin (Hb) and hematocrit (HCT) measurement before and 72 h after the operation [16].

Blood loss calculation.

Firstly, the Nadler formula was used to calculate the patient blood volume (PBV):

$${\text{PBV}}\;\left( {\text{L}} \right) = {\text{height}}\;\left( {\text{m}} \right)\;{3} \times 0.{367} + {\text{weight}}\;\left( {{\text{Kg}}} \right) \times 0.0{32} + 0.{6}0{4 }\;\left( {{\text{For}}\;{\text{ male}}\;{\text{ patient}}} \right)$$

$${\text{PBV}}\;\left( {\text{L}} \right) = {\text{height}}\;\left( {\text{m}} \right)\;{3} \times 0.{356} + {\text{weight}}\;\left( {{\text{Kg}}} \right) \times 0.0{33} + 0.{183}\; \, \left( {{\text{For }}\;{\text{female}}\;{\text{ patient}}} \right)$$

Secondly, using the Gross formula, the estimated blood loss volume (EBV) was calculated:

$$\begin{aligned} & {\text{EBV}}\;\left( {{\text{ml}}} \right) = {\text{PBV}}\;\left( {\text{L}} \right)\; \times \;\left( {{\text{Hctpre}} - {\text{Hctpost}}} \right){\text{/Hctave}} \times 1000. \\ & \left( {{\text{Preoperative}}\;{\text{ Hct}}\;{\text{ is }}\;{\text{Hctpre}},\,\;{\text{postoperative }}\;{\text{Hct}}\;{\text{ is }}\;{\text{Hctpost}},\;{\text{ and }}\;{\text{Hctave }}\;{\text{is}}\;{\text{ the }}\;{\text{average }}\;{\text{of}}\;{\text{ Hctpre }}\;{\text{and}}\;{\text{ Hctpost}}.} \right) \\ \end{aligned}$$

Thirdly, based on the Sehat formula, hidden blood loss (HBL) was calculated:

$$\begin{aligned} & {\text{HBL}}\;\left( {{\text{ml}}} \right) = {\text{EBV}}\;\left( {{\text{ml}}} \right) - {\text{VBL}}\;\left( {{\text{ml}}} \right)\; \\ & \left( {{\text{None }}\;{\text{of}}\;{\text{ patients }}\;{\text{received}}\;{\text{ blood }}\;{\text{transfusions }}\;{\text{during }}\;{\text{the }}\;{\text{surgery}},\,\;{\text{and }}\;{\text{none}}\;{\text{ drainage }}\;{\text{was }}\;{\text{placed }}\;{\text{after}}\;{\text{ both }}\;{\text{procedures}},\,\;{\text{intraoperative }}\;{\text{blood}}\;{\text{ loss }}\;{\text{could }}\;{\text{be }}\;{\text{regarded }}\;{\text{as }}\;{\text{VBL}}} \right). \\ \end{aligned}$$

The total blood loss (TBL) was finally calculated as follows:

$${\text{TBL}}\;\left( {{\text{ml}}} \right)\; = \;{\text{VBL}}\;\left( {{\text{ml}}} \right)\; + \;{\text{HBL}}\; \, \left( {{\text{ml}}} \right).\;{\text{And}}\;{\text{the}}\;{\text{percentage}}\;{\text{of}}\;{\text{HBL}} = \left( {\text{HBL/TBL}} \right)\; \times \;{1}00\% .$$

### Statistical analysis

SPSS 22.0 for Windows (SPSS Inc., Chicago, IL, USA) was used to analyze the data and statistical significance was set at *P* < 0.05 (two-sided). Descriptive statistics were shown as mean ± SD or number of cases (percentages) when appropriate. Based on baseline characteristics, it was determined whether they were comparable.

## Results

**Fig. 3:**
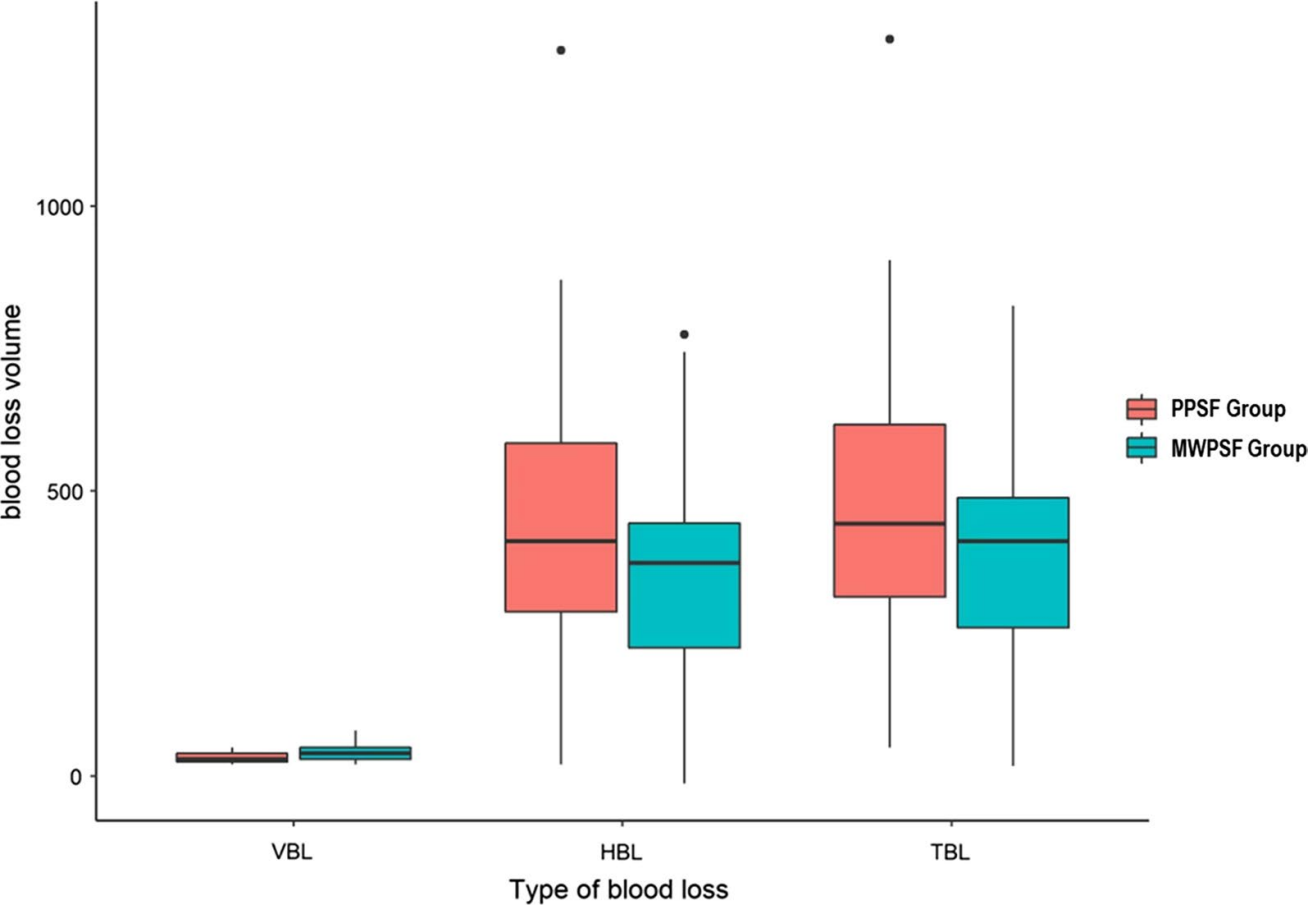
Comparison of VBL, HBL and TBL between two groups. *P* values indicate that VBL of PPSF group is significantly less than that in the MWPSF group. (*P* < 0.01) And the HBL and TBL of the PPSF group are significantly higher than that in the MWPSF group (*P* < 0.05). Abbreviation: VBL, visible blood loss; HBL, hidden blood loss; TBL, total blood loss

Table 1 shows the characteristics of patients, and the *P* values indicate that there are no significant differences between the two groups. Perioperative parameters of the patients are compared in Table 2. Compared with the PPSF group, operation time of MWPSF is shorter. The fluoroscopy times are 13.6 ± 3.0 in PPSF group and 5.6 ± 1.6 in MWPSF group (*p* < 0.001). No significant differences are detected in terms of incisions length, post-operative hospitalization time and follow up periods between the two groups. As shown in Table 3, perioperative parameters regarding hidden blood loss are recorded. As none of the patients had received blood transfusions during or after the surgery, and none drainage was placed, so intraoperative blood loss could be regarded as VBL. The intraoperative blood loss in PPSF group is 31.9 ± 9.6 ml, which is significantly less than that in the MWPSF group (44.0 ± 14.9 ml). The HBL (445.7 ± 228.9 ml), and HBL% (91.2 ± 7.7%) of the PPSF group are significantly higher than that in the MWPSF group (*P* < 0.05). And the TBL of the PPSF group (477.6 ± 228.8 ml) is also more than that in the MWPSF group (401.0 ± 171.3 ml). During the follow-up period, the postoperative complications of the two groups are recorded. (Table 4), (Fig. 3).

**Table 1:** Characteristics of the patients

	PPSF	MWPSF	*P* value
Number of patients	58	61	
Age (years)	46.0 ± 12.7	45.3 ± 11.1	0.76
Male/female	32/26	33/28	0.91
Height	166.0 ± 9.7	168.9 ± 10.0	0.11
Weight	65.1 ± 10.7	66.0 ± 12.7	0.68
BMI	23.6 ± 2.9	23.0 ± 2.8	0.29
Fracture segment			
T11	7	10	
T12	16	16	
L1	24	23	
L2	11	12	

Data are represented as mean ± SD. *P* values indicating no significant difference among two groups

**Table 2:** Perioperative parameters of the patients

	PPSF	MWPSF	*P* value
Mean operative time (min)	72.7 ± 10.2	50.7 ± 8.7	0.001
Incisions length (cm)	8.2 ± 0.5	8.1 ± 0.5	0.66
Fluoroscopy times	13.6 ± 3.0	5.6 ± 1.6	0.001
Post-operative hospitalization time (day)	3.8 ± 0.8	3.7 ± 0.8	0.85
Follow up period (month)	23.4 ± 7.3	21.1 ± 6.4	0.07

Data are represented as mean ± SD

*PPSF* percutaneous pedicle screw fixation, *MWPSF* mini-open Wiltse approach with pedicle screw fixation

**Table 3:** Perioperative parameters regarding hidden blood loss

	PPSF	MWPSF	*P* value
Intraoperative blood loss (ml)	31.9 ± 9.6	44.0 ± 14.9	0.001
Preoperative Hb values (g/L)	142.3 ± 12.7	140.6 ± 13.5	0.46
Postoperative Hb values (g/L)	122.0 ± 13.0	123.0 ± 13.5	0.67
ΔHb (g/L)	20.3 ± 6.7	17.5 ± 6.4	0.021
Preoperative Hct values (%)	42.7 ± 3.7	41.8 ± 4.0	0.22
Postoperative Hct values (%)	38.3 ± 3.8	38.0 ± 4.0	0.63
Average Hct values (%)	40.5 ± 3.6	40.0 ± 3.9	0.38
PBV (L)	4.36 ± 0.61	4.17 ± 0.74	0.14
EBV (ml)	477.6 ± 228.8	401.0 ± 171.3	0.04
VBL (ml)	31.9 ± 9.6	44.0 ± 14.9	0.001
HBL (ml)	445.7 ± 228.9	357.0 ± 170.7	0.021
TBL (ml)	477.6 ± 228.8	401.0 ± 171.3	0.04
HBL %	91.2 ± 7.7	83.7 ± 22.9	0.02

Data are represented as mean ± SD

*Hb* haemoglobin, *ΔHb* Hb difference from preoperative to postoperative, *Hct* haematocrit, *PBV* patient’s blood volume, *EBV* estimated blood loss volume, *VBL* visible blood loss, *HBL* hidden blood loss, *TBL*, total blood loss, *HBL%*, the percentage of HBL

**Table 4:** Postoperative complications of two groups

	PPSF (*n* = 58)	MWPSF (*n* = 61)
Spinal cord injury	0 (0%)	0 (0%)
Cerebrospinal fluid leakage	0 (0%)	0 (0%)
Screw misplacement	0 (0%)	0 (0%)
Infection	0 (0%)	0 (0%)
Wound complications	0 (0%)	1 (1.6%)
Deep venous thrombosis	0 (0%)	0 (0%)
Pneumonia	1 (1.7%)	0 (0%)
Urine storage	2 (3.4%)	1 (1.6%)

The postoperative complications of two groups were shown in Table 4

## Discussion

Thoracolumbar fracture is the most common type of spinal fracture due to its special anatomical structure [1, 2, 4]. Surgery at early stage is of great importance for the postoperative recovery of physiological function and the improvement of quality of life for patients [3, 4]. The traditional surgical method is to strip the paraspinal muscle through an open approach, and use the pedicle-screw fixation system to stabilize the injured vertebra and restore the height of the fractured vertebral body [4, 6]. However, traditional surgical method has many disadvantages, including large incision, excessive blood loss, delayed postoperative functional recovery, high incidence of back pain, and aggravated posterior ligament complex (PLC) injury [6–8]. An increasing number of studies have shown that minimally invasive surgery is a better choice for the treatment of neurologically intact thoracolumbar fractures [6–11]. Currently, there are two main minimally invasive treatment techniques: PPSF and MWPSF. Minimally invasive surgery has the advantages of less blood loss, shorter operation time, better soft tissue protection, shorter postoperative hospital stay, and faster functional recovery [6–9, 11].

The application of minimally invasive surgical techniques to treat thoracolumbar fractures without nerve damage has been performed since 2015 in our hospital. We observed that although the operation time was shorter and the VBL was less during the operation, a considerable number of patients still experienced postoperative anemia. Patients with spinal fractures are often injured violently and have poor tolerance for blood loss [17, 18]. Postoperative anemia may lead to insufficient organ perfusion, increase the incidence of cardiovascular and cerebrovascular complications, and increase the risk of surgical incision infection, reduce the level of recovery, thus resulting in longer hospital stay and higher cost [18–20]. We speculated that an important cause of postoperative anemia in patients treated with minimally invasive techniques may be HBL. Sehat found that there was a significant difference between the amount of visible blood loss and that calculated from the results of auxiliary examinations before and after surgery in patients with total knee arthroplasty (TKA) [12, 19]. Therefore, they proposed the concept of HBL. In recent years, the study of perioperative HBL in spine surgery has received growing attention. Studies have shown that HBL accounts for a large proportion in transforaminal lumbar interbody fusion (TLIF) surgery and PKP surgery [21–24]. Similarly, perioperative HBL in patients with lumbar fractures cannot be ignored, and postoperative indicators such as hemoglobin must be closely monitored to avoid potential adverse effects of anemia.

In our study, 119 patients with neurologically intact thoracolumbar fractures were analyzed. All of them performed minimally invasive surgery for spinal fractures in our hospital, of which 58 cases received PPSF and 61 cases received MWPSF. In this study, we used a relatively reliable method to estimate the HBL. By Nadler, Gross and Sehat formulas, we obtained the patient blood volume (PBV), estimated blood loss volume (EBV) and hidden blood loss (HBL), respectively [16]. According to the change of hematocrit, we can calculate that the average HBL of patients was 400.3 ± 205.2 ml, accounting for 87.4 ± 17.6% of TBL, indicating that HBL was abundant and accounts for a high proportion of TBL and should not be ignored in minimally invasive surgery for spinal fractures. We believe that such a high proportion of HBL may be caused by the following factors. First, because the thoracolumbar spine was cancellous bone, the injured vertebral body after fracture will have a lot of blood oozing during the perioperative period, and surgery cannot reduce this type of blood loss. Second, insufficient intraoperative hemostasis may lead to persistent postoperative bleeding. Third, hemolysis due to various causes may also lead to blood loss.

When comparing data between the PPSF and MWPSF group, we found that although the intraoperative blood loss in the PPSF group (31.9 ± 9.6 ml) was less than that in the MWPSF group (44.0 ± 14.9 ml), the TBL was higher in the PPSF group. This is because that the HBL in the PPSF group was higher than that in the MWPSF group (*P* = 0.021). We believe that there are two main reasons. First, it may because that the injuries of deep soft tissues during the process of percutaneous pedicle screws placement and connecting rod placement, resulting in a large amount of exudation in the soft tissue space after surgery. Second, for patients in the PPSF group, it was difficult to completely stop the bleeding in deep tissues during surgical process due to the limited surgical field of view. However, in the MWPSF group, because of the sufficient exposure during the surgery, the operation under direct vision can fully stop the bleeding in deep tissues, thereby reducing the HBL. Therefore, we should not ignore the large number of HBL when performing minimally invasive surgery for spinal fractures. Especially in percutaneous pedicle screw fixation, we should pay more attention to the fine operation during the surgery, protect the deep soft tissues, and use thrombin and tranexamic acid appropriately to reduce the HBL. Despite a small amount of intraoperative bleeding, the possibility of postoperative anemia should not be overlooked. Attention should be paid to HBL after surgery, and interventions such as blood transfusion should be given if necessary. In the future, we plan to further investigate the factors that contribute to the increase in HBL, in order to develop approaches to reduce perioperative HBL.

Our study has some limitations. First, as a single-center, non-randomized, retrospective study, it may be hindered by selection bias. To further evaluate HBL in minimally invasive surgery for spinal fractures, we need a prospective randomized controlled trial with adequate sample size. Second, the relatively small sample size of this study may affect statistical power. Third, the HBL in the study is calculated by the formula of Nadler, Gross and Sehat, which inevitably has a certain error compared with the actual HBL.

## Conclusion

In conclusion, in the minimally invasive surgical treatment of thoracolumbar fractures, the perioperative HBL is much higher than VBL and should not be ignored. Although PPSF has less intraoperative blood loss, it has higher TBL and HBL than those of MWPSF. Compared with MWPSF, we should pay more attention to the postoperative anemia status of patients with thoracolumbar fractures undergoing PPSF surgery, and formulate countermeasures in time to avoid a series of complications caused by postoperative anemia.
